# Properties, production, and applications of camelid single-domain antibody fragments

**DOI:** 10.1007/s00253-007-1142-2

**Published:** 2007-08-18

**Authors:** M. M. Harmsen, H. J. De Haard

**Affiliations:** 1 Institute for Animal Science and Health (ID-Lelystad) of Wageningen University and Research Centre, Edelhertweg 15, 8219 PH Lelystad, The Netherlands; 2 Ablynx N.V., Technologiepark 4, 9052 Zwijnaarde, Belgium

**Keywords:** Single-domain, Microbial production, Yeast, Glycosylation

## Abstract

Camelids produce functional antibodies devoid of light chains of which the single N-terminal domain is fully capable of antigen binding. These single-domain antibody fragments (VHHs or Nanobodies®) have several advantages for biotechnological applications. They are well expressed in microorganisms and have a high stability and solubility. Furthermore, they are well suited for construction of larger molecules and selection systems such as phage, yeast, or ribosome display. This minireview offers an overview of (1) their properties as compared to conventional antibodies, (2) their production in microorganisms, with a focus on yeasts, and (3) their therapeutic applications.

## Introduction

**Fig. 1:**
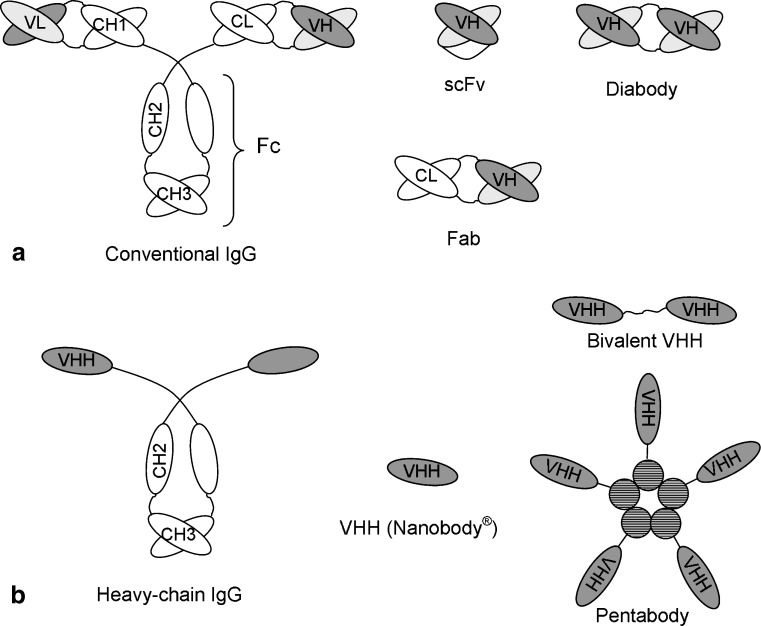
Schematic diagram of conventional (**a**) and heavy-chain (**b**) antibodies and fragments thereof. Variable domains derived from the antibody heavy (*VH*) and light (*VL*) chains are *shaded dark gray* and *light gray*, respectively, whereas constant domains (*CH* and *CL*) are not shaded. Note the absence of the light chain and CH1 domain in heavy-chain antibodies. Antibody domains that pair by noncovalent interactions are indicated by overlaying them. The B-subunits of naturally pentamerizing toxins that are used to generate pentabodies are indicated as *hatched spheres*

The field of recombinant antibody technology has rapidly progressed during the last two decades, mainly because of the interest in their human therapeutic use. The ability to select specific human antibodies by display technologies and to improve their affinity, stability, and expression level by molecular evolution has further boosted the field. Whole antibodies are complex molecules that consist of heavy and light chains (Fig. 1a). They contain an N-linked oligosaccharide attached to the second heavy-chain constant domain (CH2) that is essential for antibody effector functions such as antibody-dependent cellular cytotoxicity (ADCC), complement-dependent cytolysis (CDC), and for retaining a long serum half-life.

Although isolated antibody heavy (Utsumi and Karush 1964) and light chains (Yoo et al. 1967) can retain antigen-binding specificity, their affinity and solubility is often reduced (Ward et al. 1989). However, the paired N-terminal variable domains of heavy (VH) and light (VL) chains are sufficient for antigen binding (Sundberg and Mariuzza 2002). Such antibody fragments can be produced as monovalent antibody fragment (Fab) or as single-chain Fv (scFv) where the VH and VL domains are joined by a polypeptide linker (Fig. 1a). Their production in microbial cells is often cumbersome, especially when producing multivalent formats, because of the requirement for domain association.

The discovery that camelids (bactrian camels, dromedaries, and llamas) produce functional antibodies devoid of light chains (Hamers-Casterman et al. 1993) formed a further breakthrough because their single N-terminal domain (VHH, also referred to as Nanobody®) binds antigen without requiring domain pairing. These heavy-chain antibodies also lack the CH1 domain, which in a conventional antibody associates with the light chain and to a lesser degree interacts with the VH domain (Fig. 1b). Although single-domain antibodies later were also identified in particular cartilaginous fish (Greenberg et al. 1995), most research on the biotechnological application of single-domain antibodies was done using camelids because of their ease of handling, including immunization. Methods to isolate antigen-specific VHHs from immune (Arbabi-Ghahroudi et al. 1997; Van der Linden et al. 2000a), nonimmune (Tanha et al. 2002; Yau et al. 2003; Verheesen et al. 2006), or semisynthetic (Goldman et al. 2006) libraries using phage, yeast, or ribosome display are now well established. For further reading on these topics, we refer to recently published reviews (Muyldermans 2001; Dufner et al. 2006).

## Properties

Sequence analysis (Hamers-Casterman et al. 1993; Muyldermans et al. 1994; Vu et al. 1997; Harmsen et al. 2000) and elucidation of the crystal structure (Desmyter et al. 1996, 2001; Spinelli et al. 1996) has revealed several structural features of VHH domains. Similar to conventional VH domains, VHHs contain four framework regions (FRs) that form the core structure of the immunoglobulin domain and three complementarity-determining regions (CDRs) that are involved in antigen binding. This contrasts with shark single-domain antibodies that have a vestigial CDR2 that does not contribute to antigen binding (Streltsov et al. 2004). As compared to human VH domains, the VHH FRs show a high sequence homology of more than 80%, and their 3D structures can be superimposed (Muyldermans et al. 2001; Holliger and Hudson 2005).

The most characteristic feature of VHHs is the presence of amino acid substitutions at four FR2 positions (positions 37, 44, 45, and 47; Kabat numbering) that are conserved in conventional VH domains and that are involved in forming the hydrophobic interface with VL domains. Occasionally, antigen-binding single-domain antibody fragments that lack these characteristic FR2 substitutions are isolated from camelids. These fall into two groups. The low-affinity binders isolated from a nonimmune library originated from conventional antibodies, presumably because of the polymerase chain reaction crossover cloning artifact, as they were linked to the CH1 domain (Tanha et al. 2002). We refer to these as VHH-like conventional VHs. However, such single-domain antibody fragments with conventional-like FR2 sequences that bind antigen with high affinity are isolated from immune libraries with the high efficiency of about 10% (Conrath et al. 2001a; Saerens et al. 2004; Harmsen et al. 2005a, 2007), which equals their presence in unselected libraries (Harmsen et al. 2000). This is not expected when such clones originate from a cloning artifact. Unlike the clones isolated by Tanha et al. (2002), these clones often contain a hydrophilic residue (mostly arginine) at position 103. This substitution is probably important for their single-domain nature (Desmyter et al. 2001) because conventional antibodies contain a highly conserved hydrophobic residue (tryptophan) at this position that contacts VL. This suggests that these represent functional VHH domains derived from recombination of conventional VH gene segments with heavy-chain constant gene segments during B cell maturation. This was confirmed by the absence of the CH1 domain when such VHH domains were reisolated from the original immune repertoire using a CDR3-specific primer (De Haard, unpublished observation). Therefore, we refer to these as conventional-like VHH domains. Although the increased hydrophilicity of VHHs predominantly relies on the aforementioned changes in the former VL interface, some amino acids at positions that form a slightly hydrophobic patch on conventional VH domains that contacts CH1 (Lesk and Chothia 1988) are also changed into hydrophilic residues in VHHs (Muyldermans et al. 1994; Harmsen et al. 2000).

Furthermore, the CDRs of VHHs contain some characteristic features. Firstly, the N-terminal part of CDR1 is more variable (Vu et al. 1997; Harmsen et al. 2000; Nguyen et al. 2000). Secondly, many dromedary VHHs have an extended CDR3 that is often stabilized by an additional disulfide bond with a cysteine in CDR1 or FR2 (Muyldermans et al. 1994) resulting in the folding of the CDR3 loop across the former VL interface (Desmyter et al. 1996). A particular subfamily of llama VHHs (VHH3) also contains an extended CDR3 that is stabilized by an additional disulfide bond with a cysteine at position 50 in FR2. However, VHHs of this subfamily are rarely isolated, and most llama VHHs have CDR3 loops similar in length to those found in human VHs.

VHHs have many advantages for biotechnological applications, which are summarized in Table 1. An important advantage is their high microbial production level (see next section).

**Table 1:** Advantages of camelid single-domain antibody fragments as compared to conventional antibody fragments

Advantage	Molecular basis
Facile genetic manipulation	Single-domain nature
Increased functional size of immune libraries	No decrease in library size because of reshuffling of VL and VH domains
Facile production of multivalent formats	More flexible linker design and no mispairing of VL and VH domains
Facile production of oligoclonal preparations from single cells	No mispairing of VL and VH domains
High physicochemical stability	Efficient refolding due to increased hydrophilicity and single-domain nature
High solubility	Increased hydrophilicity
Recognition of hidden antigenic sites	Small size and extended flexible CDR3
Rapid tissue penetration, fast clearance	Small size
Well expressed	Efficient folding due to increased hydrophilicity and single-domain nature

See text for references

Several advantages result from their single domain nature. Thus, VHH libraries generated from immunized camelids retain full functional diversity. This contrasts with the diminished diversity of conventional antibody libraries because of reshuffling of VL and VH domains during library construction. As a result, high-affinity antigen-binding VHHs can be isolated by directly screening a limited number of clones from immune libraries without prior selection using display technologies (Frenken et al. 2000; Harmsen et al. 2005b). Furthermore, the single-domain nature facilitates subsequent molecular manipulation. For example, for many applications, it is advantageous to engineer monovalent antibody fragments into multivalent formats to increase functional affinity (termed avidity) or to produce bispecific antibody fragments that can simultaneously bind to different antigens. Such molecules (diabodies, Fig. 1a) can be produced using conventional recombinant antibodies using linkers between the VH and VL domains of a specific length, although this often results in aggregation and reduced affinity because of mispairing of VH and VL domains (Glockshuber et al. 1990; Whitlow et al. 1993). VHHs are more suitable for production of such formats because they allow more flexible linker design, which is important for simultaneous binding of multivalent antigens, without the problems posed by domain mispairing. Thus, several functional trivalent-bispecific VHHs have been successfully produced (Coppieters et al. 2006; Roovers et al. 2007).

The use of mixtures of a limited number of monoclonal antibodies (oligoclonal antibodies) is advantageous over single monoclonal antibodies for particular applications, such as toxin neutralization (Nowakowski et al. 2002). Because of regulatory requirements, such oligoclonals are preferentially produced from single cells. Again, VHHs are predicted to be more suitable for single-cell production of oligoclonals because of the absence of domain mispairing, although this is yet to be demonstrated experimentally.

Contrary to conventional antibodies, VHHs have been shown to remain functional at 90°C (Van der Linden et al. 1999) or after incubation at high temperatures (Van der Linden et al. 1999; Perez et al. 2001). This high apparent stability is mainly attributed to their efficient refolding after chemical or thermal denaturation and to a lesser extent because of an increased resistance against denaturation (Perez et al. 2001; Dumoulin et al. 2002; Ewert et al. 2002). The increased apparent stability is probably due to an increased hydrophilicity of the former VL interface region because a “camelized” human VH fragment that contains several of the hallmark hydrophilic amino acid residues of VHHs was more stable than the original VH fragment (Davies and Riechmann 1995, 1996), whereas “decamelization” of a VHH to mimic a VH domain reduces its thermodynamic stability (Conrath et al. 2005). In addition to these specific mutations, the packing of extended CDR3 loops against this former VL interface contributes to domain stability (Bond et al. 2003). Furthermore, refolding of VHHs only requires domain refolding, whereas conventional antibodies also require association of VH and VL domains.

VHHs can also recognize antigenic sites that are normally not recognized by conventional antibodies such as enzyme active sites (Lauwereys et al. 1998; De Genst et al. 2006) and conserved cryptic epitopes (Stijlemans et al. 2004). This facilitates their use as enzyme inhibitors or in diagnosis of trypanosome infections. The ability to recognize these recessed antigenic sites has been attributed to their smaller size and the ability of the extended CDR3 loop to penetrate into such sites (Desmyter et al. 1996; De Genst et al. 2006). It is interesting to note that this structure–function relation is also observed in a rare example of a broadly reactive human mAb that recognizes the recessed and conserved CD4-binding cavity of human immunodeficiency virus type 1 gp120 by virtue of an extended CDR3 (Zwick et al. 2003). With respect to antigen binding, the single-domain nature could be a disadvantage for binding to small antigens such as haptens and peptides because these normally bind in a groove or cavity at the VH–VL interface (Sundberg and Mariuzza 2002). Indeed, llamas immunized with clenbuterol developed conventional but not heavy-chain antibodies against this hapten (Lange et al. 2001). However, hapten- and peptide-binding VHHs have been successfully isolated using strong selection systems (Spinelli et al. 2000; Yau et al. 2003; Alvarez-Rueda et al. 2007; Harmsen et al. 2007). The affinities of VHHs are generally comparable to those of conventional antibody fragments (Muyldermans et al. 2001). Occasionally, VHHs with affinity constants (*K*_D_) as low as 100 pM are isolated (Saerens et al. 2004; De Genst et al. 2006; Harmsen et al. 2006), which equals the affinity ceiling proposed for natural antibodies (Sundberg and Mariuzza 2002).

Because of their small size of about 15 kDa, VHHs rapidly pass the renal filter, which has a cutoff of about 60 kDa, resulting in their rapid blood clearance. In addition, the small size results in a fast tissue penetration. This is advantageous for targeting of VHHs coupled to toxic substances to tumors (Cortez-Retamozo et al. 2004), in vivo diagnosis using imaging, and treatment of snake bites (Harrison et al. 2006). However, for other therapeutic applications, such as treatment of infectious or inflammatory diseases, the short serum half-life of about 2 h (Cortez-Retamozo et al. 2002; Harmsen et al. 2005a) is a disadvantage.

## Production in microorganisms

Although a fully active nonglycosylated IgG was recently produced at high level in *Escherichia coli*, most functional complete antibodies can only be efficiently produced using mammalian cells, especially when their appropriate glycosylation is required for therapeutic applications. However, antibody fragments that lack the Fc with its N-linked oligosaccharide are preferably produced in microbial systems (Arbabi-Ghahroudi et al. 2005). These have a shorter development time from gene to product and require simple well-established fermentation conditions that can be performed on large-scale resulting in costs of goods that can be as low as $1 per gram heterologous protein (Estell 2006). Most large-scale microbial production systems are based on *E. coli*, yeasts, or filamentous fungi. Production in *E. coli* can be done by secretion into the oxidizing periplasmic space or expression in the reducing cytosol. The latter requires the often cumbersome refolding of antibody fragments (Arbabi-Ghahroudi et al. 2005). Using eukaryotic microbial hosts, antibody fragments are generally produced by targeting to the secretory pathway. This enables efficient disulfide bond formation, addition of N-linked oligosaccharide, and secretion of soluble, correctly folded product to the culture medium.

VHHs have often been produced in *E. coli* (Arbabi-Ghahroudi et al. 1997; Rahbarizadeh et al. 2005). There is only one example of VHH production in filamentous fungi, which resulted in limited proteolytic degradation of the secreted product (Joosten et al. 2005) because of the high levels of proteases secreted by filamentous fungi (Gerngross 2004). VHHs have also often been produced in the yeast *Saccharomyces cerevisiae* (Frenken et al. 2000; Thomassen et al. 2002; Van der Vaart 2002). VHH production by the favored yeast expression host *Pichia pastoris* was only recently described (Rahbarizadeh et al. 2006). Occasionally, yeast-produced VHHs are N-glycosylated (Frenken et al. 2000; Harmsen et al. 2005a). This can affect antigen binding (Van der Vaart et al. 2006). Furthermore, it could complicate their therapeutic use because the addition of yeast-specific high-mannose oligosaccharides results in a high immunogenicity and decreased serum half-life because of binding to specific mannose receptors on cells of the reticulo-endothelial system (Sethuraman and Stadheim 2006).

Although VHHs are generally well produced in microorganisms, the production level of different clones can vary by a factor of 100 (Frenken et al. 2000; Harmsen et al. 2005b; Van de Laar et al. 2007). Several VHH sequence patterns can be associated with their production level. First, the presence of a potential N-linked glycosylation site often increases production levels in yeast (Sagt et al. 2000). Second, in our experience (Harmsen, unpublished observations), conventional-like VHHs are generally produced at reduced levels in yeast. This contrasts with the reported efficient production in *E. coli* of VHH-like VHs (Tanha et al. 2002) but is consistent with the increased production level of “camelized” conventional VH domains in *E. coli* (Davies and Riechmann 1995). Third, the presence of unpaired C-terminal cysteines reduces expression levels (Simmons et al. 2006). Fourth, replacement of hydrophobic residues of conventional VH domains normally interacting with CH1 increased scFv production in *E. coli* (Nieba et al. 1997), suggesting that the hydrophilic mutations that naturally occur at these positions in VHHs also contributes to their high expression level. However, there are many examples of VHHs that differ by only a few amino acids and are produced at highly variable levels where the exact amino acid change responsible for the difference in production level is difficult to predict (Frenken et al. 2000; Harmsen et al. 2005b). Furthermore, without such knowledge, VHH production can be improved by random molecular evolution using deoxyribonucleic acid shuffling (Van der Linden et al. 2000b), as has often been done for conventional antibody fragments (Dufner et al. 2006). The high refolding capability of VHHs, which is a consequence of their sequence, has also been correlated with a high production level in *E. coli* (Jespers et al. 2004; Olichon et al. 2007).

In addition to the nature of the VHH, host factors affecting VHH production have been identified. In baker’s yeast, the specific VHH production rate is correlated with growth rate (Thomassen et al. 2005) and can be up to fivefold increased by growing on ethanol as the carbon source (Van de Laar et al. 2007). Supplementation of the medium with sorbitol, casamino acids, or ethylenediamine tetraacetic acid improves VHH production by *P. pastoris* (Rahbarizadeh et al. 2006).

In addition to monovalent VHHs, several expression formats for the production of VHH multimers have been described (Fig. 1b). These include genetic fusions of two (Conrath et al. 2001b; Harmsen et al. 2005a) or three VHHs (Coppieters et al. 2006; Roovers et al. 2007) that either recognize different antigens or the same repeating antigen to increase functional affinity. Although such VHH fusions are less efficiently produced than their monovalent versions, their production level exceeds that of their conventional-antibody-based fusion counterparts without aggregation or low solubility. However, antigen binding by the C-terminal VHH in such fusions can be compromised (Conrath et al. 2001b) presumably because of steric hindrance by the N-terminal VHH. The avidity of VHHs has also been strongly increased using genetic fusions to the B-subunits of an *E. coli* toxin that self-assembles into a homopentamer (Zhang et al. 2004), resulting in pentameric recombinant antibodies (“pentabodies,” Fig. 1b).

VHHs on their own cannot recruit effector functions such as ADCC and CDC. This limits their therapeutic application. Although such effector functions can be indirectly recruited using bispecific (conventional) antibody fragments binding to host immunoglobulin (Holliger et al. 1997), it may be more efficient to recruit these functions by fusing VHHs to host Fc domains. Production of such functional antibodies requires the correct glycosylation of the CH2 domain, which until recently could only be accomplished using higher eukaryotic cells (Nguyen et al. 2003) but not by microbial production. However, this may now be feasible using *P. pastoris* strains with an engineered glycosylation machinery that are able to produce proteins with a specific human glycoform (Hamilton et al. 2006). Furthermore, transgenic mice containing hybrid llama/human antibody loci that contain llama V regions and human D, J, and C regions have recently been used to generate human heavy-chain antibodies in mice (Janssens et al. 2006).

## Therapeutic applications

Although VHHs are highly suited for applications that require a high stability, such as use in shampoo for the prevention of dandruff (Dolk et al. 2005), as capturing reagents in immunoaffinity purification (Verheesen et al. 2003), or use in biosensors (Pleschberger et al. 2004), we would like to focus on their therapeutic applications, which are more challenging. Several VHHs are now being studied for use in various disease areas, including oncology (Revets et al. 2005) and in infectious, inflammatory, and neurodegenerative diseases (Table 2).

**Table 2:** Examples of therapeutic applications of camelid VHHs

Disease	Pathogen	Target antigen	VHH valency for disease target	Additional fusion partner	Reference
Sleeping sickness	Trypanosomes	VSG oligomannose	Monovalent	Apolipoprotein L-I	Baral et al. 2006
Infant diarrhea	Rotavirus	Unknown	Monovalent	None	Van der Vaart et al. 2006
Infant diarrhea	Rotavirus	Unknown	Monovalent	*Lactobacillus* cell-surface anchor	Pant et al. 2006
Piglet diarrhea	*E. coli*	F4 fimbriae	Monovalent	None	Harmsen et al. 2006
Caries	*S. mutans*	I/II adhesion	Monovalent	None	Kruger et al. 2006
FMD	FMD virus	VP1	Monovalent	PEG	Harmsen et al. 2007
Sepsis	*N. meningitidis*	LPS	Monovalent	None	El Khattabi et al. 2006
Cancer	–	CEA	Monovalent	β-Lactamase	Cortez-Retamozo et al. 2004
Cancer	–	EGF receptor	Bivalent	Anti-albumin VHH	Roovers et al. 2007
Rheumatoid arthritis	–	TNFα	Bivalent	Anti-albumin VHH	Coppieters et al. 2006
Brain disorders	–	α (2,3)-Sialoglycoprotein	Monovalent	None	Muruganandam et al. 2002
Neurodegenerative diseases	–	Bax	Monovalent	None	Gueorguieva et al. 2006

VHHs are especially suited for oral immunotherapy because of their resistance against extremes of pH and the capacity to bind to the target at high concentrations of chaotropic agents (Dumoulin et al. 2002, 2003). Administration to piglets of a VHH that effectively prevents intestinal attachment of *E. coli* bacteria that cause diarrhea resulted in poor in vivo protection (Harmsen et al. 2005b) because of degradation by gastrointestinal proteases (Harmsen et al. 2006). However, by selection for proteolytic stability, a VHH could be isolated from the original library that was not degraded in vivo (Harmsen et al. 2006). VHHs that successfully prevented diarrhea caused by rotavirus in a mouse model were similarly selected for resistance against the acidic environment of the stomach (Van der Vaart et al. 2006). Alternatively, VHH proteolysis can be prevented by local VHH production using natural gut commensal bacteria. Thus, diarrhea could also be prevented by lactobacilli that produce rotavirus-neutralizing VHHs fused to a cell surface anchor (Pant et al. 2006). Treatment of caries, caused by *Streptococcus mutans*, with VHHs conferred only limited protection (Kruger et al. 2006). Because these VHHs should function in the oral cavity, the low level of protection cannot be due to proteolytic VHH degradation within the gastrointestinal tract.

The short serum half-life because of a rapid renal clearance limits the efficacy of VHHs in many parenteral applications. Therefore, VHHs have been targeted to normally long-lived serum proteins such as albumin (Coppieters et al. 2006; Roovers et al. 2007) or immunoglobulin (Harmsen et al. 2005a) using bispecific VHHs recognizing these serum proteins in addition to the therapeutic target, resulting in half-lives that equal the half-life of albumin (2 days in mice) and immunoglobulin (9 days). An alternative well-known approach to increase serum half-life of proteins is the chemical addition of polyethylene glycol (PEG). Such PEGylation of foot-and-mouth disease (FMD) virus-neutralizing VHHs not only increased serum half-life but also increased in vitro neutralizing potency to levels above that of the hyperimmune serum (Harmsen et al. 2007). However, in contrast to the full protection afforded by the hyperimmune serum, these VHHs poorly protected guinea pigs from FMD viral challenge infection, suggesting that Fc-mediated effector functions are required for efficient in vivo protection (Harmsen et al. 2007).

Nevertheless, many diseases were successfully treated with VHHs in the absence of Fc-mediated effector functions. These VHHs either are used as targeting devices for toxic enzymes or block a specific molecular interaction. For example, sleeping sickness was successfully treated with VHHs that bind to a trypanosome coat protein and were fused to the apolipoprotein L-1 enzyme, resulting in trypanosome lysis (Baral et al. 2006). In oncology, a VHH directed against carcinoembryonic antigen was used for targeting the genetically fused β-lactamase to tumor cells. This enzyme then converts an injected nontoxic prodrug into a toxic drug in the vicinity of the targeted tumor cells, leading to their killing (Cortez-Retamozo et al. 2004). Several VHH therapies are also being developed for treatment of oncology or inflammatory diseases based on blocking molecular interactions. VHHs binding to epidermal growth factor receptor (EGFR) can block epidermal growth factor (EGF) binding to its receptor, which can be used to treat solid tumors (Roovers et al. 2007). Tenfold more potent EGFR-binding VHHs could be obtained by construction of bivalent formats. It is interesting to note that the recently approved conventional antibody Panitumumab directed against EGFR also blocks EGF binding and is expected to give poor ADCC and CDC (Reichert and Valge-Archer 2007). Furthermore, by blocking receptor interaction, VHHs binding to tumor necrosis factor-α can be used for treatment of rheumatoid arthritis (Coppieters et al. 2006). The potency of bivalent formats was 500-fold increased as compared to monovalent VHHs and even exceeded the potency of clinically used conventional antibodies both in vitro and in a murine arthritis model. Similarly, lipopolysaccharide (LPS)-binding VHHs were isolated that block LPS binding and signaling to host cells for treatment of sepsis (El Khattabi et al. 2006).

The potential immunogenicity of VHHs could compromise their parenteral therapeutic use, especially in treatments that require repeated injections. Until now, multiple injections of VHHs have not shown any immunogenicity in mice, as assessed by the presence of specific antibodies, T cell proliferation, or cytokine levels (Cortez-Retamozo et al. 2002; Coppieters et al. 2006). This could rely on their high sequence homology to conventional VH domains and on their high stability because aggregation of proteins is known to increase immunogenicity (Hermeling et al. 2004). If necessary, technologies developed to decrease immunogenicity of mouse monoclonal antibodies (Presta 2006) could also be applied to VHHs. Alternatively, immunogenicity could be reduced by the use of conventional-like VHHs, which have an even higher structural homology to conventional VH domains.

For their use in targeting drugs across the blood–brain barrier (BBB) into the brain, VHHs were selected that transmigrate the human BBB in an in vitro model and accumulate in the brain after intravenous injection into mice (Muruganandam et al. 2002). These could be used for treatment of neurological disorders. Finally, Bax-specific VHHs have been expressed in the cytoplasm, resulting in so-called intrabodies, to prevent oxidative-stress-induced apoptosis that is implicated in several neurodegenerative diseases (Gueorguieva et al. 2006). Because of their stability, VHHs are especially suited for intrabody production because this requires expression in the reducing environment of the cytoplasm (Gueorguieva et al. 2006; Rothbauer et al. 2006).

## Conclusions

Since the discovery of heavy-chain antibodies in 1993, the field of single-domain antibody fragments has been rapidly growing. VHHs have many advantages for biotechnological applications. They can be economically produced in microorganisms and have a high stability. Furthermore, they are highly suited for expression as multivalent, including bispecific, formats or as enzyme fusions. This permits a plug-and-play approach, where, depending on the target, biology potency can be increased by multivalent constructs or bispecific VHH recognizing two different targets can be made. This also enables the tailor-made design of serum half-life using site-directed PEGylation or by targeting to long-lived serum proteins using bispecific VHHs. Although fusions of targeting VHHs to Ig-binding VHHs or Fc can be used to recruit effector functions most current research on VHHs focuses on therapeutic applications where such effector functions are not required. Finally, conventional whole antibodies occasionally give side effects because of their bivalent nature, which can result in target cross-linking, or the presence of the Fc region. Evidently, such side effects are not expected to occur using monovalent VHHs. This, however, is yet to be confirmed as the first VHH has entered phase I clinical trials in 2007 (http://www.ablynx.com).
